# Interpreting nonverbal cues to deception in real time

**DOI:** 10.1371/journal.pone.0229486

**Published:** 2020-03-09

**Authors:** Josiah P. J. King, Jia E. Loy, Hannah Rohde, Martin Corley

**Affiliations:** 1 Department of Psychology, PPLS, University of Edinburgh, Edinburgh, Scotland, United Kingdom; 2 Centre for Language Evolution, PPLS, University of Edinburgh, Edinburgh, Scotland, United Kingdom; 3 Department of Linguistics and English Language, PPLS, University of Edinburgh, Edinburgh, Scotland, United Kingdom; State University of New York Downstate Medical Center, UNITED STATES

## Abstract

When questioning the veracity of an utterance, we perceive certain non-linguistic behaviours to indicate that a speaker is being deceptive. Recent work has highlighted that listeners’ associations between speech disfluency and dishonesty are detectable at the earliest stages of reference comprehension, suggesting that the manner of spoken delivery influences pragmatic judgements concurrently with the processing of lexical information. Here, we investigate the integration of a speaker’s gestures into judgements of deception, and ask if and when associations between nonverbal cues and deception emerge. Participants saw and heard a video of a potentially dishonest speaker describe treasure hidden behind an object, while also viewing images of both the named object and a distractor object. Their task was to click on the object behind which they believed the treasure to actually be hidden. Eye and mouse movements were recorded. Experiment 1 investigated listeners’ associations between visual cues and deception, using a variety of static and dynamic cues. Experiment 2 focused on adaptor gestures. We show that a speaker’s nonverbal behaviour can have a rapid and direct influence on listeners’ pragmatic judgements, supporting the idea that communication is fundamentally multimodal.

## Background

In natural communication, speakers can convey information via multiple channels. Along with spoken delivery, a speaker’s gestures, postures and facial expressions can all offer extra-linguistic information about the speaker or message. Listeners can be affected by such information in a number of ways. They may, for example, make inferences about the speaker’s emotion [1, 2]. Alternatively, their interpretation of the message itself may change, for example if extra-linguistic information causes them to believe that the speaker is being dishonest [3]. The present paper focuses on this latter circumstance. In particular, we investigate whether, and how, speakers’ postures or adaptor gestures (e.g., fidgeting movements) affect listeners’ judgements of veracity.

This is especially relevant in light of recent work investigating the manner in which utterances are spoken. Work focusing on the auditory modality has established an association between spoken disfluency and deceit that emerges from the early stages of comprehension. Loy et al. [4] used a visual world eye and mousetracking paradigm in which participants were presented with images of two objects, and heard utterances describing the location of some treasure purportedly hidden behind one of the objects. These utterances were presented as having been elicited in a previous experiment, in which the speaker was known to have been lying some of the time. Crucially, Loy et al. [4] manipulated the manner of spoken delivery, with half of the experimental items containing a speech disfluency. Participants were tasked with clicking on the object they *believed* to be concealing the treasure, choosing either the object named in the utterance (indicating a judgement of honesty), or a distractor (dishonesty). They were more likely to judge disfluent utterances as dishonest than fluent ones (as indicated by a greater probability of clicking on the distractor in a disfluent trial). Importantly, disfluency resulted in an early bias in both eye and mouse movements towards the not-referred-to object. This suggests that speech disfluency is already incorporated into listeners’ ideas concerning deceptive speech, and has an immediate effect on their interpretation of an utterance.

Turning from the auditory to the visual modality, research suggests that many nonverbal aspects of delivery are associated by listeners with deception. In an analysis of 33 studies, Zuckerman and DePaulo [3] found that nine out of the ten visual cues to deception that were investigated were believed to be indicative of deceit. In 13 studies reporting relationships between cues and subsequent deception judgements (rather than explicit beliefs about cues), three (smiling, gaze, and postural shifts) of the four available visual cues were associated with perceived dishonesty. However, links between nonverbal behaviour and perceived deception have been studied only in terms of after-the-fact judgements, or by assessing listeners’ explicit beliefs about cue validity [5, 6]. How and when these cues are incorporated into judgements of deception remains unclear.

Research suggests that information presented in speakers’ hand movements is integrated into language comprehension along a similar time course as the processing of speech [7, 8]. However, this research has tended to focus on the comprehension of the semantic content of iconic gestures (movements which visually represent content): For instance, iconic gestures which are incongruent with sentential context have been associated with electrophysiological responses which are similar in latency, amplitude, and topography to those elicited when the incongruency is presented in speech [7].

To our knowledge, however, no studies to date have explored the time course of how a speaker’s body language informs the pragmatic interpretation of their message. This may be because a speaker’s nonverbal behaviours are substantially more varied than speech hesitations: For a listener, they may serve both as potential markers of metacognitive states and planning processes, and as an alternative modality in which the speaker conveys semantic information [9, 10]. Any process linking a speaker’s movements with deception must be subtle enough to discriminate types of nonverbal behaviours, or risk over-attribution by labelling irrelevant cues as signs of deceit. Furthermore, listeners associate static visual cues with deception (for instance, eye-gaze [6]), suggesting that judgements of deception are not linked just to variations in movement, but to an array of nonverbal cues.

**Fig 1:**
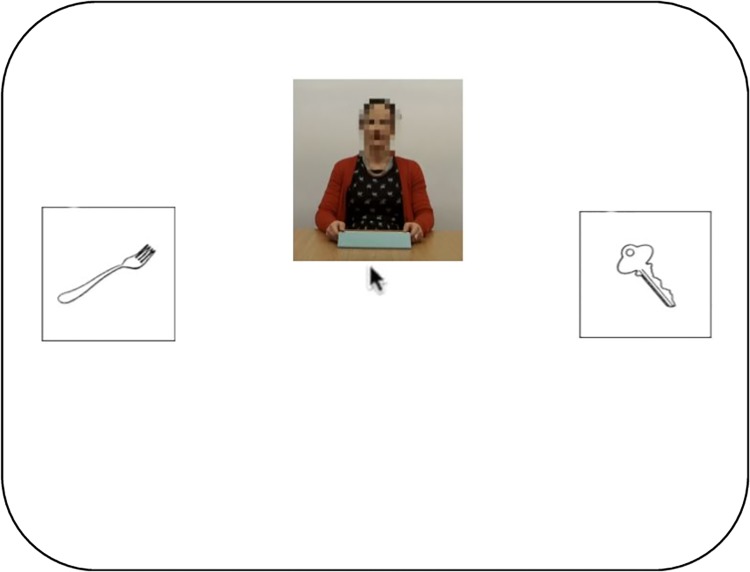
Layout of experimental display: Visual-world-paradigm with video stimulus.

Here, we aim to shed light on the question of how visual information about a speaker is integrated into the pragmatic interpretation of language, by investigating whether the time course of listeners’ judgements of deception are influenced by nonverbal behaviours in a similar way to hesitations and other auditory aspects of the manner of speech. The two experiments presented here extend the ‘treasure game’ paradigm from Loy et al. [4] to include a video of a potentially deceptive speaker describing the location of some treasure purportedly hidden on the screen (behind one of two objects; Fig 1). Crucially, we manipulate the presence or absence of potential visual cues to deception in the video. Listeners attempt to guess, and click on, the true location of the treasure, which allows us to infer whether they believe the speaker to be lying or telling the truth. If listeners associate a given visual cue with deception, then following these cues they should be more likely to click on the object which has not been mentioned. By measuring listeners’ eye and mouse movements as the speaker’s descriptions unfold, we can investigate their interpretations of what is being said over time.

In Experiment 1, we focus on how trunk movements (visible movements of the torso forward, backward or sideways) influence judgements of deception, with filler trials presenting two further types of nonverbal behaviour (different static postures, and adaptor gestures—movements directed towards the self or objects, often considered to be aimed at improving comfort or reducing stress, e.g., fidgeting or adjusting clothing). The justification of our focus on trunk movements is twofold: (a) Previous research indicates that listeners perceive these movements as cues to lying [3, 5], and (b) they are a plausible utterance-initial gesture [11], allowing us to ensure that gestures can be viewed in their entirety before visual targets are referred to. Based on a post-hoc analysis of filler trials which suggested that listeners’ judgements were in fact most strongly influenced by the speaker’s adaptor gestures, we designed Experiment 2 to replicate this latter effect.

## Experiment 1

Experiment 1 makes use of eye and mouse tracking to investigate whether a speaker’s nonverbal behaviours affect a listener’s judgements of deception over time. The experiment was presented as a ‘lie detection game’. Each trial included a video and audio recording of a potentially deceptive speaker describing the location of some hidden treasure. Throughout a trial, two images, depicting potential treasure locations, remained visible on the screen. Participants were tasked with using the mouse to click on the object they believed to be concealing the treasure. Critical trials presented videos of the speaker either producing a trunk movement immediately prior to utterance playback, or sitting motionless (no cue) for the equivalent amount of time. Our aim was to investigate whether and when these nonverbal cues would be associated with falsehood. To increase the variety of the speaker’s nonverbal movements, we included filler trials presenting videos of the speaker sitting in a different posture or producing an adaptor gesture, alongside those of the speaker producing no cue.

### Ethics statement

Ethical approval for this research was obtained from the Ethics Committee of Philosophy, Psychology and Language Sciences, University of Edinburgh, in accordance with the guidelines of the British Psychological Society. Participants gave written consent to take part in the study and were informed of their right to withdraw at any time. The individual presented in the experiment materials has given written informed consent (as outlined in PLOS consent form) to use their image.

### Participants

Twenty-four self-reported native speakers of English were recruited from the University of Edinburgh community, and took part in the experiment in return for a payment of £4. Participants all had normal or corrected-to-normal vision, and were all right-handed mouse users.

### Materials

Visual and audio stimuli were taken from Loy et al., [4]. Visual stimuli consisted of 120 line drawings from Snodgrass and Vanderwart [12], sixty of which served as the object named as hiding the treasure (referents) and the other sixty as distractors. Referents were randomly paired with distractors and presented across sixty trials (20 critical trials and 40 fillers). As in Loy et al. [4], critical referents and distractors were matched for both ease of naming (H < 1.0) and familiarity (F ≤ 3.0). Each pair of referents was associated with an audio recording of fluent speech specifying the image as the object that the treasure was hidden behind (“The treasure is behind the <referent>”).

To create the video recordings to use with the previously-recorded audio stimuli, we recorded a volunteer repeating the phrase “the treasure is behind the <object>” while either sitting motionless or performing a given gesture (trunk movement, different static posture, adaptor gesture). Videos showed the speaker in front of a plain white background, seated at a table upon which rested a tablet computer (on which the referent, distractor, and treasure were purported to be displayed). The face shown in each video was pixelated, to allow different videos to be associated with different audio recordings without providing evidence that the visual and auditory channels had been recorded separately.

In 20 critical trials, the audio recordings were paired with 10 videos showing the speaker producing no cue, and five different videos of trunk movements (each used in two different critical trials). Critical trials were counterbalanced across two lists, such that audio recordings paired with a motionless speaker in one list were paired with trunk movements in the other. Forty filler trials were added to each list. These trials presented participants with 10 videos showing no cue (each used in two different filler trials); 10 videos showing the speaker motionless but in a different posture; and 10 videos showing the speaker producing an adaptor gesture (e.g., fidgeting, tapping fingers on table, scratching chin). The pairings of videos with pairs of images (and associated audio tracks) in filler trials were randomly assigned for each run of the experiment. All videos are available on the Open Science Framework (OSF) at https://osf.io/gu3dp/.

We identified a timepoint in each video recording at which, according to our judgement, it would be natural for audio to begin. For videos showing a trunk movement, this was the frame of the video at which the movement ended, meaning that there was no overlap between the gestural cue and the ensuing speech. The time to audio onset was matched in videos showing no cue, thus controlling for any sensitivity to the duration of video prior to speech. For videos showing an adaptor gesture the amount of overlap between the visual cue and speech varied according to the experimenters’ judgements of what appeared natural; time to audio onset was matched in videos showing the speaker in different static postures.

### Procedure

The experiment was presented using OpenSesame version 3.1 [13]. Stimuli were displayed on a 21 in. CRT monitor with a resolution of 1024 × 768, placed 850 mm from an Eyelink 1000 Tower-mounted eye tracker which tracked eye movements at 500 Hz (right eye only). Audio was sampled at 44100 Hz and presented in stereo from speakers on either side of the monitor. Videos were presented at 25 frames per second, and mouse coordinates were sampled at every frame. Eye movements, mouse coordinates and object clicked (referent or distractor) were recorded for each trial.

**Fig 2:**
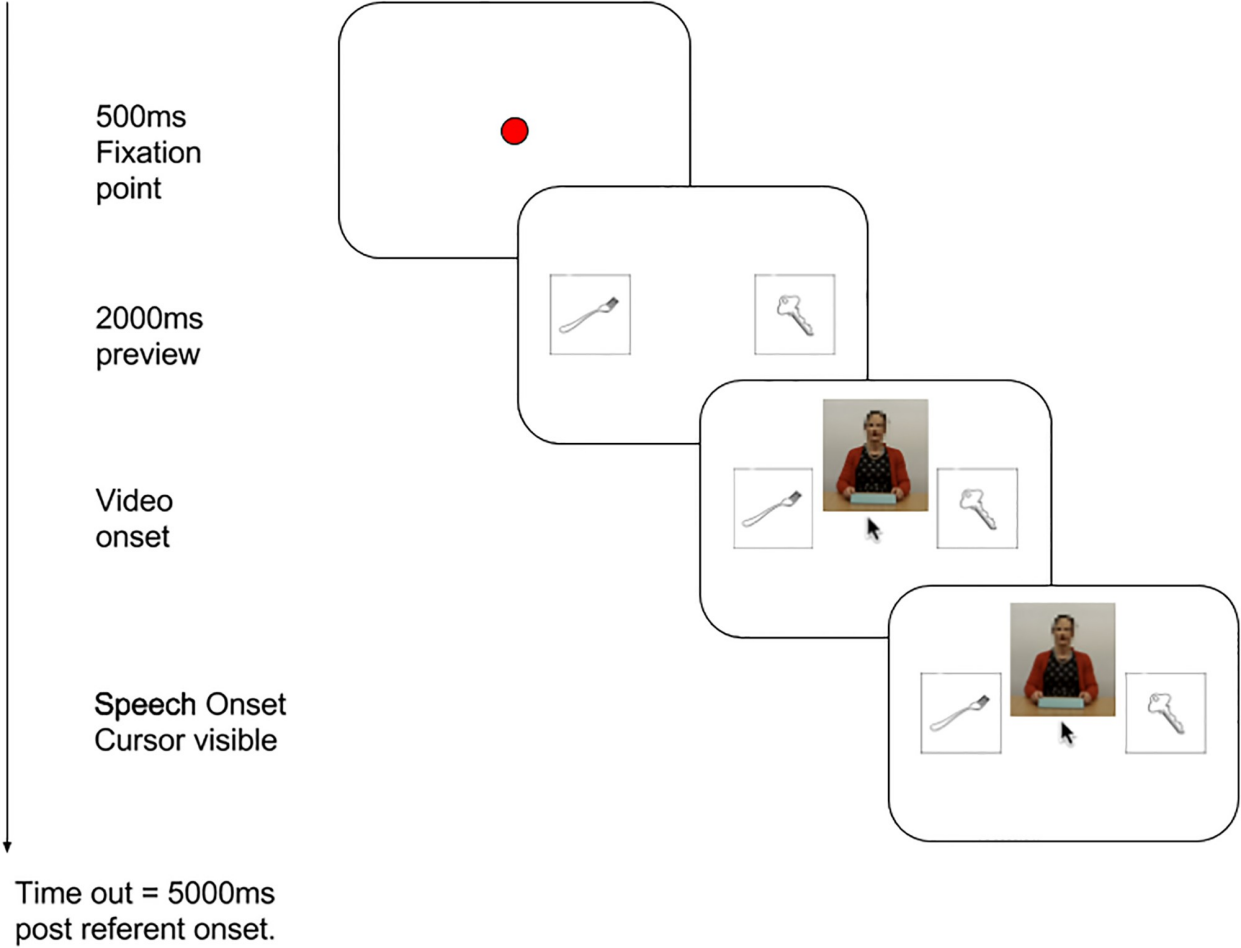
Procedure of a given trial, Experiments 1 and 2.

Fig 2 represents a sample trial from the experiment. Between trials, participants underwent a manual drift correction to ensure accurate recordings from the eye tracker. After this, the central fixation dot turned red for 500ms to signify progression to the trial. This was replaced by two images corresponding to the referent and distractor, each measuring 150 × 150 pixels. These were centred vertically and positioned such that the centre of the left and right image was 15% from the corresponding edge of the display. The positions (left vs. right) of referents and distractors were randomly chosen, with the constraint that for each participant, referents occurred equally often on each side, separately for critical and filler trials. 2000 ms after the onset of the image display, a video was added to the screen, and the mouse pointer was centred and made visible. The video, measuring 266 × 284 pixels, was displayed with the bottom edge at the vertical midpoint of the screen and centred horizontally. Playback of the audio recording began at the assigned frame of the video (see materials above). The trial ended once the participant clicked on either object, or timed out 5000 ms after onset of the referent noun, at which point participants saw a message telling them to click on subsequent objects faster.

Participants were told that they were watching recordings taken from a previous experiment, in which one participant was tasked with describing the location of some hidden treasure with the aim of misleading another participant into choosing the wrong location. To emphasise this, the instructions included a photograph of two people purportedly participating in this previous experiment. Participants were told that the speakers in the previous experiment had lied approximately half of the time. Participants were instructed to click on the object behind which *they believed* the treasure to be hidden, with the overall aim of accumulating as much treasure as they could across the experiment. Participants received no feedback after their object clicks, except on bonus trials, which are described in the next section. They were told that the top scorers would be able to enter their names on a high-score table, which was shown at the beginning of the experiment.

The order of trials was randomly assigned on each run of the experiment. Participants completed five practice trials (one of which was presented as a bonus trial) prior to the main experiment. Two of these presented a video showing no cue, two displayed a video of the speaker in different postures, and one displayed a video of the speaker making a trunk movement.

### Bonus trials

To maintain motivation throughout the study, participants were told that there were a number of ‘hidden bonus rounds’ which offered more treasure than regular rounds. 25% of filler trials (half including a gestural cue; half presenting a video showing no cue) were randomly designated as bonus rounds for each participant. These trials were visually identical to regular trials. However, following the mouse click (regardless of the object chosen), a message was displayed informing participants that they had successfully located bonus treasure.

### Post-test questionnaire

Participants were asked to complete a short post-test questionnaire which asked whether they had noticed anything odd about the visual or audio stimuli. Any participant who indicated that they had noticed anything unusual was then questioned further, to decide whether they believed that the speech and gesture had been produced naturally and simultaneously. All participants were subsequently debriefed, during which they were told that the audio and video were created separately and stitched together, and asked again verbally if they had noticed anything unusual in that respect. Responses to the questionnaire and debrief were used to determine whether participants should be excluded from the analysis.

## Results

### Analysis

Data from four participants who indicated suspicion of the supposed origins of the audiovisual stimuli based on the post-test questionnaire and/or debrief were removed from all analyses, leaving data from twenty participants. Of the resultant 400 critical trials, one trial, in which the participant did not click on either the referent or distractor, was excluded from all analyses. Analysis was carried out in R version 3.6 [14].

Eye- and mouse-tracking analysis was conducted on the 2000 ms following referent-noun onset. This window extends just beyond the average time it took participants to click the mouse on either object (mean = 1805 ms). Initial analyses (pre-registered for Experiment 2 on the Open Science Framework) of eye- and mouse-tracking data used linear mixed effects regression to model the difference in empirical logit transformed proportions (see [15]) of fixations and cumulative mouse movements towards one object over the other. Following previous work [4, 16], this analysis was conducted on the initial 800 ms window following referent onset. During the review process, a number of improvements were suggested, resulting in the methods described below. Code and results for both initial and final analysis are available on the OSF, along with explanation of the decisions leading to this change (https://osf.io/m4ehd/).

Eye fixation data was averaged into 20 ms bins (of 10 samples), and ordinal generalised additive mixed models (GAMMs) fit with the mgcv package version 1.8-28 [17, 18] were used to model the object on which participants fixated in a given bin. Ordinal GAMMs can be used to assess the probability that an outcome *y* takes a value from *r* = 1, …, *R*, with *r* being labels for ordered categories (for specific advantages of GAMMs over ordinal regression, see [19]). In the context of the current experiment, this corresponds to the probability that the object fixated upon took a value from *r* = distractor, neither, referent. A fixation bias towards the referent over the distractor will thereby be reflected in the probability of *y* falling into a category higher up this ordinal scale (in which referent > neither > distractor).

Mouse-tracking analysis was conducted analogously to eye-tracking. The position of the mouse was sampled every frame of the video (presented at 25 frames per second). Variability in processing speed of the experiment script resulted in this equating to the position of the cursor being recorded approximately every 38 ms (mean = 38.18, SD = 2.83). Using the *X* coordinates only, we calculated the number of screen pixels moved and the direction of movement (towards either referent or distractor), and the cumulative distance travelled towards each object in each bin. Movements beyond the outer edge of either object were considered to be ‘overshooting’ and were not included in calculations (1.78% of samples). Ordinal GAMMs were used to model the object (referent > neither > distractor) towards which the cursor had moved the most distance by each bin.

Initial ordinal generalised additive models included intercepts for condition and *ordered factor difference smooths* (using thin plate regression splines) of time between conditions (reference level: No cue). In mgcv, ordered factor difference smooths fit *K* − 1 centered smooths (where 1, …, *k* are the levels of the factor), in which each smooth models the difference between the smooth for the reference level and the *k*-th level of the factor. This enables us to investigate how the probability of which object is fixated upon (or towards which the cursor has moved most) over time differs between experimental conditions (the nonverbal cue shown in the video).

In GAMMs, shrunk factor smooths can be used as non-linear equivalents of by-participant and by-item random intercepts and slopes in linear mixed models (see, e.g., [20]). To preserve power, the optimal random-effects structure was determined using model selection (see [21]): To assess whether the additional complexity of random-effects was warranted, the compareML function in the itsadug package (version 2.3, [22]) was used to perform a *χ*^2^ test of fREML scores of models with and without a) non-linear random smooths (including an intercept shift) of time for each item and each participant and subsequently b) by-item and by-participant random effects of condition. The best-fit model for both eye and mouse movements included non-linear random smooths (including an intercept shift) of time for each item and each participant and by-item and by-participant random effects of condition.

Object clicks (referent over distractor) were modelled using mixed effects logistic regression fitted using the blme package (version 1.0.4, see [23]), with a fixed effect of condition (nonverbal behaviour in the video: No cue vs. trunk movement, dummy coded with no-cue as the reference), by-participant and by-item random intercepts and by-participant slopes of condition. Time taken to click an object (measured from referent onset) was log transformed and modelled using mixed effects linear regression using the lme4 package (version 1.1.21, [24]) with fixed effects of condition (no cue vs. trunk movement, dummy coded with no-cue as the reference) and object clicked (referent vs. distractor, deviation coded) and by-participant and by-item random intercepts and by-participant slopes of condition.

### Eye movements

**Fig 3:**
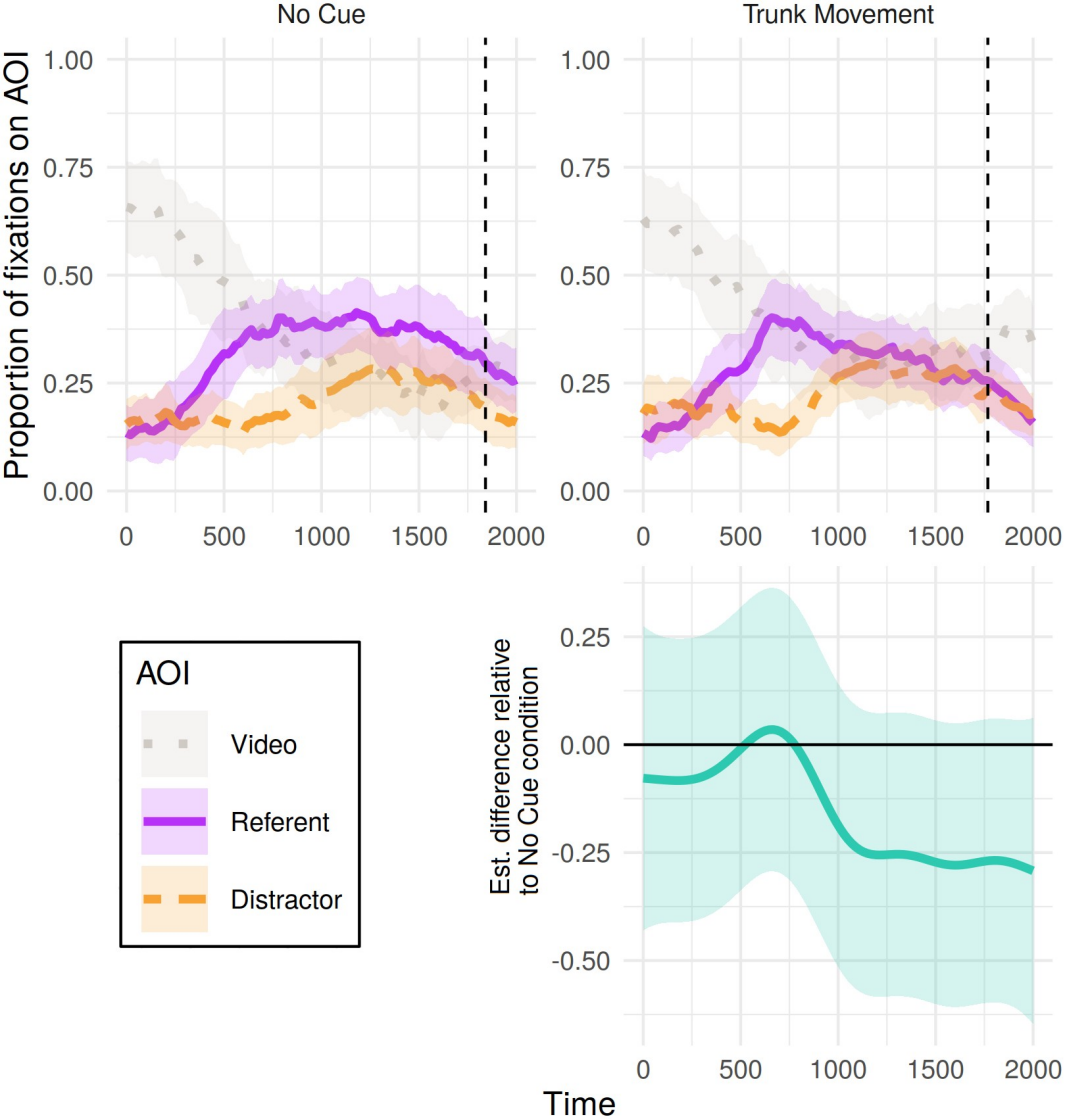
Eye tracking results for critical trials in Experiment 1: Proportion of fixations to each object (referent or distractor) and the video, from 0 to 2000 ms post-referent noun onset, calculated out of the total sum of fixations for each 20 ms time bin. Shaded areas represent 95% confidence intervals derived via bootstrapping subject data (R = 1000). Vertical dotted lines indicate mean click time by condition. Bottom panel presents the estimated difference between non-linear smooths comparing each condition to the no-cue condition (negative value indicates fewer fixations to referent > neither > distractor in the cue condition). Pointwise 95%-confidence intervals are shown by the shaded bands—where these do not overlap with zero, the difference between conditions is significant.

Fig 3 shows the time course of fixations to referents, distractors and videos in critical trials for the 2000 ms from referent onset, split by condition (whether the video showed no cue or a trunk movement). Analysis of the object fixated (referent > neither > distractor) over this 2000 ms window revealed a non-linear difference over time between conditions (Table 1). The bottom panel of Fig 3 shows the combined effect of the linear and non-parametric differences in objects fixated on between trunk movement and no-cue trials. Values above zero indicate a greater probability that the object of fixation is higher up the ordinal scale (referent > neither > distractor) in the trunk movement condition relative to the no-cue condition. Conversely, values below zero correspond to a greater probability of the object of fixation falling lower down this scale following a trunk movement. As presented in Fig 3, differences between conditions are in the expected direction (greater probability of fixating distractor following a trunk movement than no cue), however, these differences are small, not reaching statistical significance.

**Table 1:** Eye-tracking model results, Experiment 1: Results from ordinal generalised additive mixed model of object fixated in a given bin (referent > neither > distractor).

*Parametric coefficients*	*Estimate*	*Std. Error*	*t-value*	*p-value*
(Intercept)	0.52	0.15	3.49	<.001
Trunk Movement	-0.16	0.16	-0.95	.34
*Smooth terms*	*edf*	*Ref.df*	*F-value*	*p-value*
s(Time)	3.66	3.99	1.85	.114
s(Time):Trunk Movement	5.33	6.40	6.00	<.001
s(Time, Participant)	125.49	179.00	236.34	<.001
s(Time, Item)	120.41	179.00	73.66	<.001
s(Participant, Condition)	34.27	38.00	29.50	<.001
s(Item, Condition)	32.47	38.00	19.66	<.001

### Mouse movements

**Fig 4:**
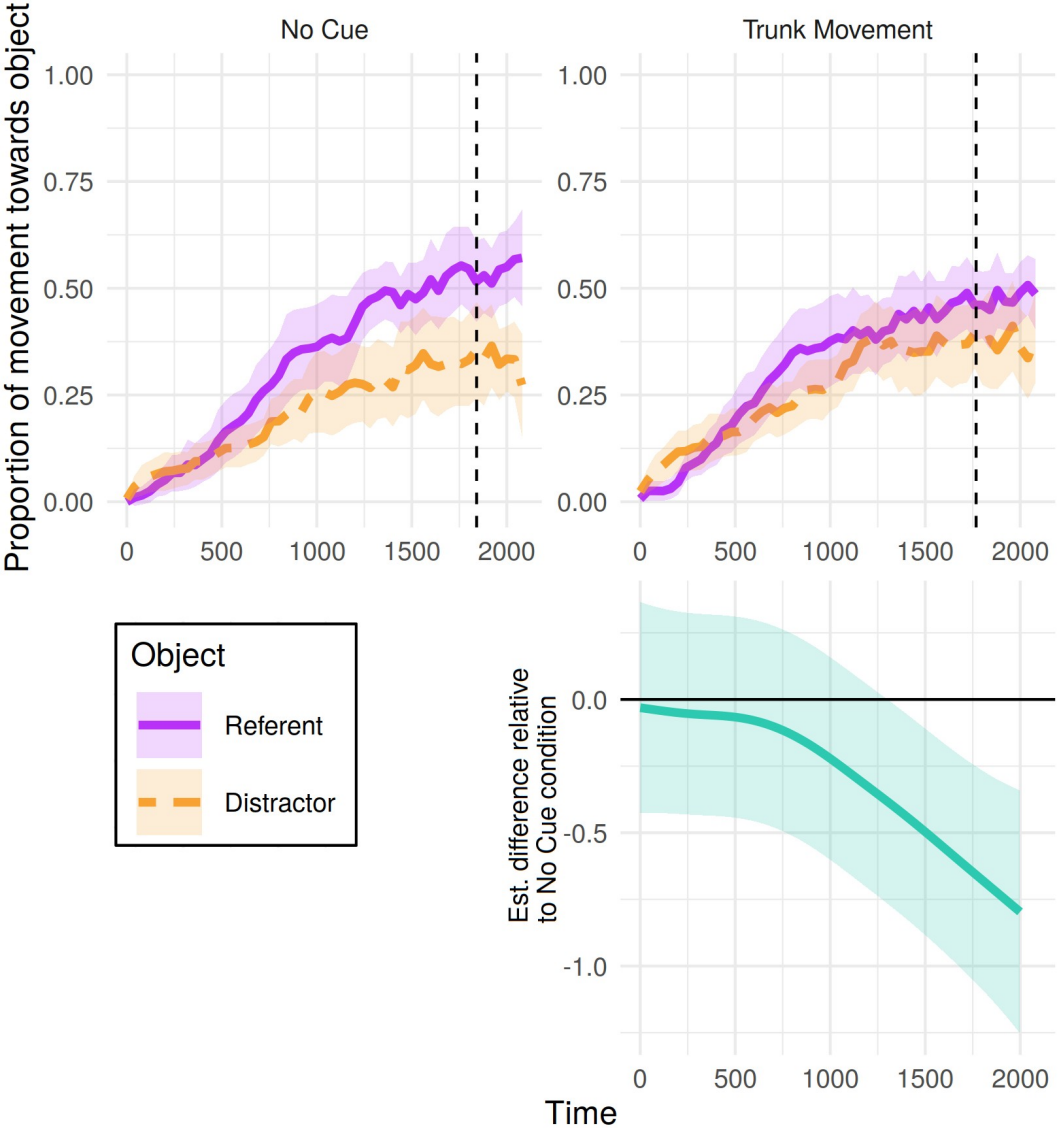
Mouse tracking results for critical trials in Experiment 1: Proportion of cumulative distance travelled toward each object from 0 to 2000 ms post-referent onset. Proportions were calculated from the total cumulative distance participants moved the mouse until that time bin. Shaded areas represent 95% confidence intervals derived via bootstrapping subject data (R = 1000). Vertical dotted lines indicate mean click time by condition. Bottom panel presents the estimated difference between non-linear smooths comparing each condition to the no-cue condition (negative value indicates fewer mouse movements towards referent > neither > distractor in the cue condition). Pointwise 95%-confidence intervals are shown by the shaded bands—where these do not overlap with zero, the difference between conditions is significant.

Fig 4 shows the time course of the proportions of cumulative distance the mouse moved towards the referent and distractor in critical trials for the 2000 ms period from referent onset, split by whether the video showed either no cue or a trunk movement. Analysis of the object (referent > neither > distractor) towards which the cursor travelled most cumulative distance over this window revealed a non-linear difference over time between conditions (Table 2). Compared to eye-movements, a clearer difference between conditions was evident in participants’ mouse-movements: From around 1300 ms after referent-noun onset, trunk movements were associated with a greater probability of participants’ having moved the cursor less towards the referent and more towards the distractor than in the no-cue condition (Fig 4, bottom panel).

**Table 2:** Mouse-tracking model results, Experiment 1: Results from ordinal generalised additive mixed model of object towards which the cursor had moved most up to that time bin (referent > neither > distractor).

*Parametric coefficients*	*Estimate*	*Std. Error*	*t-value*	*p-value*
(Intercept)	0.62	0.20	3.13	.002
Trunk Movement	-0.31	0.19	-1.62	.105
*Smooth terms*	*edf*	*Ref.df*	*F-value*	*p-value*
s(Time)	1.00	1.00	1.27	.26
s(Time):Trunk Movement	2.51	3.10	13.27	<.001
s(Time, Participant)	135.10	179.00	31.33	.18
s(Time, Item)	97.60	179.00	28.99	<.001
s(Participant, Condition)	20.06	38.00	8.24	<.001
s(Item, Condition)	31.61	38.00	14.21	<.001

### Object clicks

Participants clicked on the referent in 56% of critical trials and the distractor in 44%. Table 3 shows the numbers of clicks across all participants to either object split by whether the video showed no cue or a trunk movement. Participants were more likely to click on the referent than the distractor following a video showing no cue (OR = 1.77, 95% CI = [1.12, 2.82], *p* = .015). Marginal reductions of this bias following videos of the speaker producing a trunk movement (OR = 0.56, 95% CI = [0.30; 1.06], *p* = .074, Table 4), and in the time taken to click the mouse (*β* = −0.06, 95% CI = [−0.12, 0.01], *t* = −1.74, Table 5) are compatible with an account that participants’ judgements were influenced by the presence of trunk movements. However, as for eye-tracking results, the evidence is weak.

**Table 3:** Objects clicked in critical trials in Experiment 1: Clicks recorded on each object (referent or distractor) split by condition (no cue vs. trunk movement).

*Condition*	*Clicks to Referent*	*Clicks to Distractor*
No Cue	125 (62.5%)	75 (37.5%)
Trunk Movement	99 (49.7%)	100 (50.3%)

**Table 4:** Objects clicked model results, critical trials Experiment 1: Mixed effects logistic regression model results of mouse clicks to referent over distractor.

*Predictor*	*Odds Ratio*	*95% CI*	*p-value*
(Intercept)	1.77	1.12–2.82	.015
Trunk Movement	0.56	0.30–1.06	.074
Var(1|Participant)	0.54		
Var(Trunk Movement|Participant)	1.18		
Var(1|Item)	0.08		

**Table 5:** Time-to-click model results, critical trials Experiment 1: Mixed effects linear regression model results of log transformed times taken to click the mouse.

*Predictor*	*Estimate*	*95% CI*	*t-value*
(Intercept)	7.47	7.38–7.56	165.52
Trunk Movement	-0.06	-0.12–0.01	-1.74
Clicked Distractor	-0.05	-0.01–0.11	1.72
Var(1|Participant)	0.03		
Var(Trunk Movement|Participant)	0.10		
Var(1|Item)	<0.01		

## Additional analyses of filler trials

In the post-test verbal questioning, 8 participants (40%) specifically mentioned responding to the speaker’s hand-movements in their judgements of whether or not the speaker was deceptive. We conducted analyses on filler trials to investigate whether the types of nonverbal behaviours presented in these trials (different postures and adaptor gesturing) were influencing participants’ judgements of deception. Analysis of filler trials was conducted on 797 trials (3 trials were excluded from analysis due to no mouse click on either object), with nonverbal behaviour comprising three levels: No cue, different posture and adaptor gesture (dummy coded in all analyses, again with ‘no cue’ as the reference).

Analyses followed the same procedure as for critical trials, with *χ*^2^ tests of fREML scores indicating that the same model structure (non-linear random smooths of time for each item and each participant and by-item and by-participant random effects of condition) for eye and mouse tracking analyses was warranted. To avoid issues of model non-convergence, analysis of object clicks and time-to-click included random intercepts by-participant and by-item, but no random slopes.

### Eye movements

**Fig 5:**
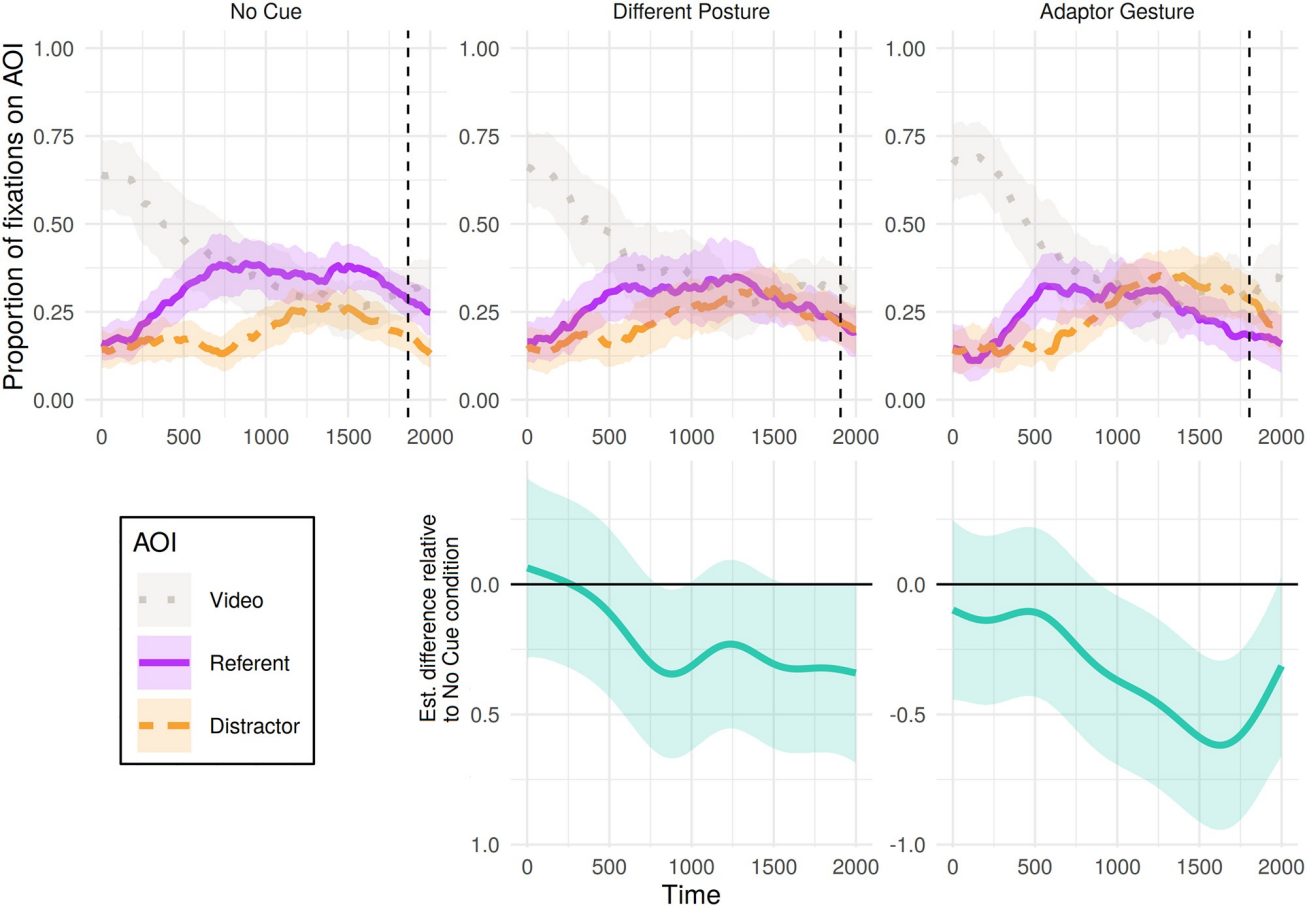
Eye tracking results for filler trials in Experiment 1: Proportion of fixations to each object (referent or distractor) and the video, from 0 to 2000 ms post-referent onset, calculated out of the total sum of fixations for each 20 ms time bin. Shaded areas represent 95% confidence intervals derived via bootstrapping subject data (R = 1000). Bottom panel presents the estimated difference between non-linear smooths comparing each condition to the no-cue condition (negative value indicates fewer fixations to referent > neither > distractor in the cue condition). Pointwise 95%-confidence intervals are shown by the shaded bands—where these do not overlap with zero, the difference between conditions is significant.

Fig 5 shows the time course of proportions of fixations to referent, distractor and video split by the type of nonverbal behaviour shown in the filler trials. Analysis revealed non-linear differences in participants’ object fixations over time between both cue conditions and the no-cue condition (Table 6). Difference curves between each condition and the no-cue condition (Fig 5, bottom panel) indicate a difference in participants’ fixations following videos of adaptor gestures (relative to no-cue videos) emerging approximately 900 ms post referent-noun onset. Following this point, the object of participants’ fixations was more likely to be lower down the scale of referent > neither > distractor in comparison to the no-cue condition. For the condition in which videos presented the speaker in a different static posture, differences in fixations from the no-cue condition are less clear but in the expected direction, comparable to effect of trunk movements in the critical trials.

**Table 6:** Eye-tracking model results, filler trials in Experiment 1: Results from ordinal generalised additive mixed model of object fixated in a given bin (referent > neither > distractor).

*Parametric coefficients*	*Estimate*	*Std. Error*	*t-value*	*p-value*
(Intercept)	0.61	0.14	4.41	<.001
Adaptor Gesture	-0.34	0.16	-2.09	.037
Different Posture	-0.21	0.16	-1.33	.184
*Smooth terms*	*edf*	*Ref.df*	*F-value*	*p-value*
s(Time)	4.58	4.99	2.38	.0363
s(Time):Adaptor Gesture	5.62	6.73	17.57	<.001
s(Time):Different Posture	5.47	6.58	10.11	<.001
s(Time, Participant)	129.62	179.00	418.49	<.001
s(Time, Item)	255.14	179.00	167.30	<.001
s(Participant, Condition)	53.64	57.00	86.14	<.001
s(Item, Condition)	107.35	116.00	41.28	<.001

### Mouse movements

**Fig 6:**
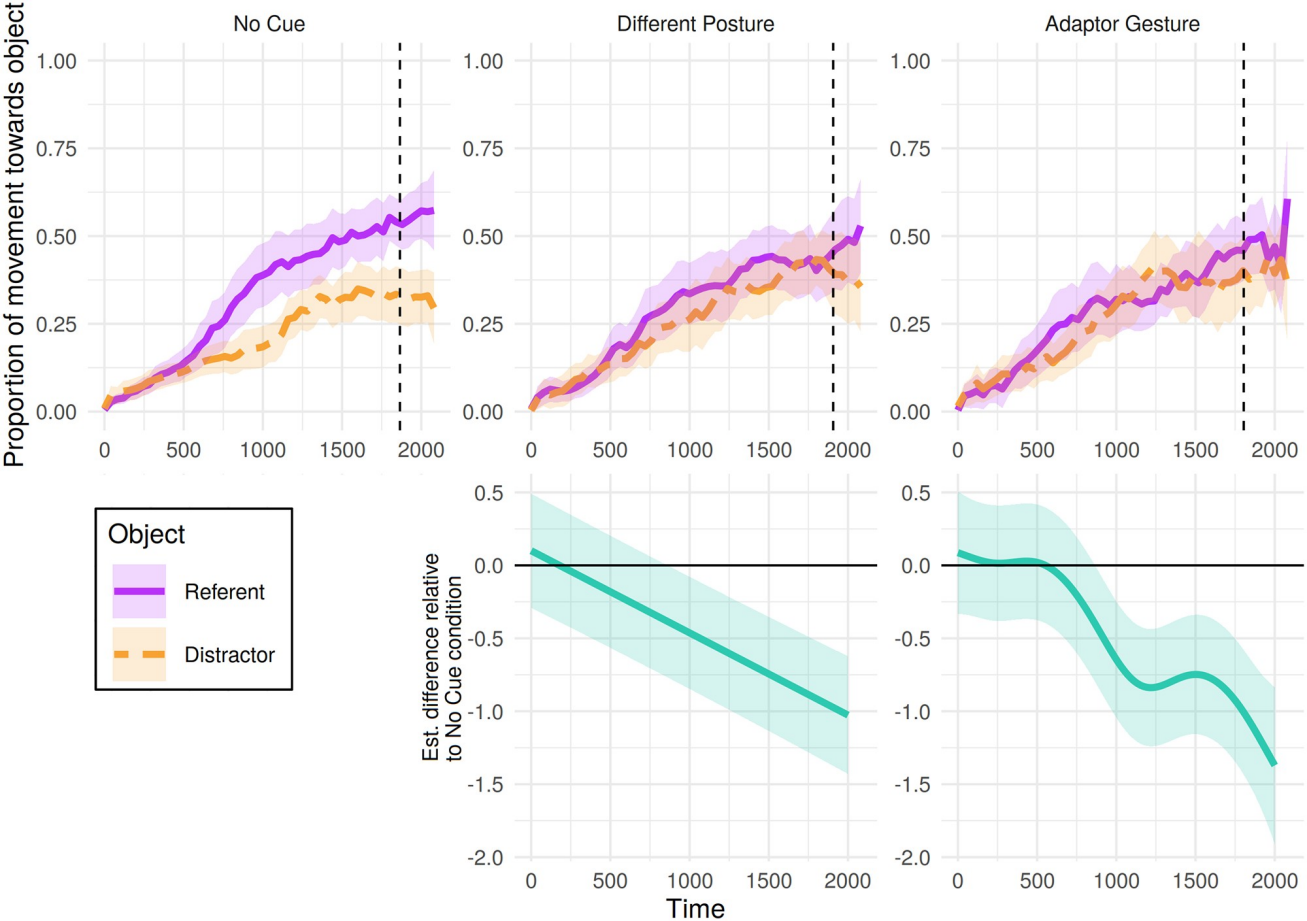
Mouse tracking results for filler trials in Experiment 1: Proportion of cumulative distance travelled toward each object from 0 to 2000 ms post-referent onset. Proportions were calculated from the total cumulative distance participants moved the mouse until that time bin. Shaded areas represent 95% confidence intervals derived via bootstrapping subject data (R = 1000). Bottom panel presents the estimated difference between non-linear smooths comparing each condition to the no-cue condition (negative value indicates fewer mouse movements towards referent > neither > distractor in the cue condition). Pointwise 95%-confidence intervals are shown by the shaded bands—where these do not overlap with zero, the difference between conditions is significant.

Fig 6 shows participants’ mouse movements towards the referent and distractor split by the type of nonverbal cue shown in the filler trials. Patterning with their fixation preferences, analysis of the object towards which participants’ moved the mouse the most cumulative distance over this window revealed non-linear differences over time between both cue conditions and the no-cue condition (Table 7). Difference curves for each condition relative to the no-cue condition (Fig 6, bottom panel) indicate these differences appearing at a similar time as in eye-movements.

**Table 7:** Mouse-tracking model results, filler trials in Experiment 1: Results from ordinal generalised additive mixed model of object towards which the cursor had moved most up to that time bin (referent > neither > distractor).

*Parametric coefficients*	*Estimate*	*Std. Error*	*t-value*	*p-value*
(Intercept)	0.90	0.20	4.44	<.001
Adaptor Gesture	-0.51	0.20	-2.60	.009
Different Posture	-0.48	0.20	-2.46	.014
*Smooth terms*	*edf*	*Ref.df*	*F-value*	*p-value*
s(Time)	1.00	1.00	9.26	.002
s(Time):Adaptor Gesture	5.83	6.92	30.37	<.001
s(Time):Different Posture	1.01	1.01	121.71	<.001
s(Time, Participant)	128.21	179.00	78.88	.419
s(Time, Item)	251.75	359.00	46.54	.411
s(Participant, Condition)	41.11	57.00	37.19	<.001
s(Item, Condition)	79.70	116.00	15.56	<.001

### Object clicks

Table 8 shows the numbers of clicks in filler trials across all participants to either object, split by the type of nonverbal behaviour presented in the video. For trials in which the video showed a speaker producing no cue, participants tended to click on the referent rather than the distractor (OR = 1.84, 95% CI = [1.43, 2.36], *p* < .001). For trials in which the videos showed the speaker either in a different posture or producing an adaptor gesture, this bias to click on the referent was reduced (OR = 0.50, 95% CI = [0.35, 0.71], *p* < .001 and OR = 0.38, 95% CI = [0.27, 0.55], *p* < .001 respectively), suggesting that presence of these types of nonverbal cues influenced participants’ final judgements of whether the speaker was truthful or dishonest (Table 9). Similar to trunk movements, the presence of adaptor gesture cues (but not different static posture cues) was marginally associated with a reduction in the time participants took to click the mouse (*β* = −0.04, 95% CI = [−0.09, 0.01], *t* = −1.76, Table 10).

**Table 8:** Objects clicked in filler trials in Experiment 1: Clicks recorded on each object (referent or distractor) split by each type of nonverbal behaviour presented in the video.

*Condition*	*Clicks to Referent*	*Clicks to Distractor*
No Cue	256 (64.5%)	141 (35.5%)
Different Posture	96 (48.0%)	104 (52.0%)
Adaptor Gesture	83 (41.5%)	117 (58.5%)

**Table 9:** Objects clicked model results, filler trials in Experiment 1: Mixed effects logistic regression model results of mouse clicks to referent over distractor in filler trials.

*Predictor*	*Odds Ratio*	*95% CI*	*p-value*
(Intercept)	1.84	1.43–2.36	<.001
Different Posture	0.50	0.35–0.71	<.001
Adaptor Gesture	0.38	0.27–0.55	<.001
Var(1|Participant)	0.06		
Var(1|Item)	0.08		

**Table 10:** Time-to-click model results, filler trials Experiment 1: Mixed effects linear regression model results of log transformed times taken to click the mouse.

*Predictor*	*Estimate*	*95% CI*	*t-value*
(Intercept)	7.48	7.40—7.57	168.34
Different Posture	-0.00	-0.05—0.05	0.04
Adaptor Gesture	-0.04	-0.09—0.01	-1.76
Clicked Distractor	0.03	-0.01—0.08	1.58
Var(1|Participant)	0.03		
Var(1|Item)	0.01		

## Discussion

Experiment 1 investigated how the pragmatic inferences listeners make about a speaker’s honesty are influenced by the presence of nonverbal cues to deception, in the form of trunk movements. We presented videos of a potentially deceptive speaker making a statement about the location of some treasure. We measured the eye and mouse movements made by participants who were tasked with clicking on one of two possible treasure locations: one which was mentioned, and one which was not. Participants were thus making implicit decisions about the honesty of each utterance.

As in previous studies using versions of this paradigm [4, 16], participants showed a tendency to interpret an utterance as truthful (as indicated by more clicks to the named object) when there was no obvious cue to deception (i.e., speaking fluently or sitting motionless). The presence of a trunk movement prior to speech onset had only a marginal influence on participants’ judgements of deception, as evidenced by the objects selected, in contrast to the existing literature [3, 5]. Patterning with mouse-clicks, participants’ unfolding preferences to fixate on—and move the mouse towards—different objects in the display may have been influenced by the presence of a trunk movement in the video, but again these effects were weak, especially in eye-movements. Furthermore, more reliable differences found in mouse movements emerged relatively late after the referent-noun onset (as opposed to the effect of speech disfluency within 800 ms seen in previous studies [4, 16]). Together, these results suggest that listeners may have, at best, weakly associated trunk movements with deception (as reflected in their goal-oriented mouse-movements).

In contrast, additional analyses of filler trials suggested that participants may have been influenced by the other types of nonverbal behaviour presented in the experiment: Videos showing the speaker producing either an adaptor gesture or sitting in a different posture were associated with a greater likelihood of judgements of deception than videos showing the speaker producing no cue. Furthermore, the influence of one of these nonverbal cues—adaptor gestures—was notably evident in both eye and mouse movements, appearing early in the time-course, in line with previous research.

However, the filler trials differed from experimental trials in three important ways. First, referents were not counterbalanced; any findings may have partially or wholly reflected differences between the plausibilities of particular objects as treasure locations. Second, the longest duration of a referent-noun used in filler trials was approximately 1100 ms, rather than 800 ms for critical referents (which provided the reasoning for analyses in previous studies considering only the initial 800 ms following referent-noun onset; [4, 16]). This allows for a later influence of gesture than of disfluency, rendering direct comparison between modalities difficult. Third, since the trials under consideration here were filler trials, 25% of the items analysed were identified immediately after the mouse click as bonus trials; this may have reinforced any associations participants formed between particular gestures and the speaker’s perceived honesty as a result of feedback they received.

From a practical viewpoint, participants’ eye and mouse movements in Experiment 1 support the compatibility of the visual world paradigm with a range of video stimuli: Viewing videos in which movements co-occurred with speech (e.g., adaptor gestures) did not prevent the emergence of a fixation bias (e.g., the bias to fixate the referent within the first 500 ms in Fig 5, top right panel). Moreover, with many adaptor gesture cues occurring within only a small area of the video (such as finger tapping), the results suggest that many comparatively discrete gestures may be salient enough to elicit comprehension effects (e.g., on final judgements of deception). However, the nonverbal behaviours that appeared to have the greatest influence on participants’ judgements were never the intended focus of Experiment 1, and these trials differed from critical trials in a number of respects.

In addition to highlighting the salience of hand movements in making deception judgements, responses to the post-test questioning revealed that 4 participants (20%) claimed to rely on ‘how relaxed the speaker looked’ in making their judgements, with two of these specifically mentioning that the videos in which the speaker produced no cue presented her in an unrelaxed posture. It is possible that the association between nonverbal behaviour and deception is driven by perceived anxiety. In this case, our findings are largely in keeping with the literature, in that adaptor gestures, but not shifts of posture, have been suggested to be associated with nervousness [2]. With this in mind, and given that the effects of adaptor fillers in Experiment 1 were larger than those of posture changes, we designed Experiment 2 as a more controlled investigation of the association between adaptor gesturing and perceived dishonesty. New video stimuli were created to ensure that recordings showed the speaker either producing a typically nervous adaptor gesture, or sitting motionless and in a relaxed posture. There were no filler trials.

## Experiment 2

Using the same paradigm as Experiment 1, participants in Experiment 2 heard utterances accompanied by a video of a speaker either producing an adaptor gesture or sitting motionless, and were tasked with making an implicit judgement on whether the speaker was lying or telling the truth.

The videos used in Experiment 2 showed adaptor gestures which have previously been suggested to be associated with anxiety [2], and were pre-tested for perceived nervousness in the speaker. As a manipulation check, after the treasure-game task, participants were asked to rate how nervous the speaker looked in each video (without audio).

### Participants

Twenty-three self-reported native English speaking participants took part in exchange for £3 compensation.

### Materials

The 40 images used in critical trials in Experiment 1 (20 referents; 20 distractors) were used across twenty trials. As in Experiment 1, these images were displayed in referent-distractor pairs, with each pair shown alongside a recorded utterance naming the referent as the location of the treasure. The pairing of referents and distractors on each trial was randomised.

As in Experiment 1, each pair of images and recorded utterance was presented alongside a video clip of a person purported to be the speaker of the utterance. Twenty-eight new video clips were recorded (18 different adaptor gestures; 10 no-cue). Care was taken to ensure that the videos including no cue showed the speaker in a relaxed posture. Adaptor gestures were based on descriptions of anxious nonverbal behaviour from Gregersen [2]. All 28 videos were pre-tested for perceived nervousness of the speaker. Ten native English speakers, who did not take part in either of Experiments 1 or 2, were told that they were going to watch videos (without audio) of someone being questioned in a stressful situation. They were asked to rate how nervous the speaker looked in each video (1: very relaxed, 7: very nervous). The 10 videos showing adaptor gestures with the highest ratings for nervousness (Mean = 4.1, SD = 1.5) were included in the experiment, along with the 10 videos showing no cue (Mean = 1.9, SD = 1.1) (available on the OSF at https://osf.io/59vax/).

The 20 referents were counterbalanced across two lists such that each referent that occurred with a video showing adaptor gesturing in the first list occurred with a video showing no cue in the second. The pairings of referents with specific videos within each condition was randomised for each run of the experiment.

### Procedure

The experimental procedure matched that of Experiment 1 in all aspects with the exception of the following changes. First, the size of the video stimuli changed slightly to 236 × 336 pixels, due to videos being recorded in a different room and cropped accordingly to include only the plain background and the speaker. Second, the duration of video presented prior to audio playback was fixed at 1400 ms (after the initiation of gestural cues in all videos) in order to control for participants interpreting the duration from video to speech onset as speech initiation time and in turn associating this with deceit. This was possible as we did not constrain nonverbal cues to be fully presented prior to speech (as we did for trunk movements in Experiment 1). Third, because there were no fillers, we did not include any ‘bonus’ trials, so participants did not receive any feedback during the experiment.

After the main task, participants were asked to watch all 20 videos again, without audio, and asked to rate how nervous they thought the speaker looked (using the 1–7 scale described above). Participants then completed the same post-test questionnaire as in Experiment 1, with data being excluded from analysis on the same basis.

## Results

### Analysis

Analysis was conducted on data from 20 participants (Data from three participants was excluded on the basis of responses to post-test questionnaire). We followed the same analysis strategy as that used for the critical trials in Experiment 1. Of the 400 recorded trials, those which did not result in a click to either object (3) were excluded from analyses. The mouse position was recorded approximately every 38 ms (mean = 37.66, SD = 0.99), and 1.27% of samples were removed due to being beyond the outer edge of either object. The best fitting eye and mouse-tracking models had the same parametric and smooth terms as for the analyses of critical trials in Experiment 1.

Participants’ post-test ratings (1–7) of how nervous the speaker appeared in each video were analysed using mixed effects linear regression with fixed effects of nonverbal behaviour (no cue vs. adaptor gesturing), by-video and by-participant random intercepts and a by-participant random slope of nonverbal behaviour. Results confirmed that videos of gesturing were perceived as more nervous than videos showing no cue (*β* = 3.20, *SE* = 0.32, *t* = 10.08).

### Eye movements

**Fig 7:**
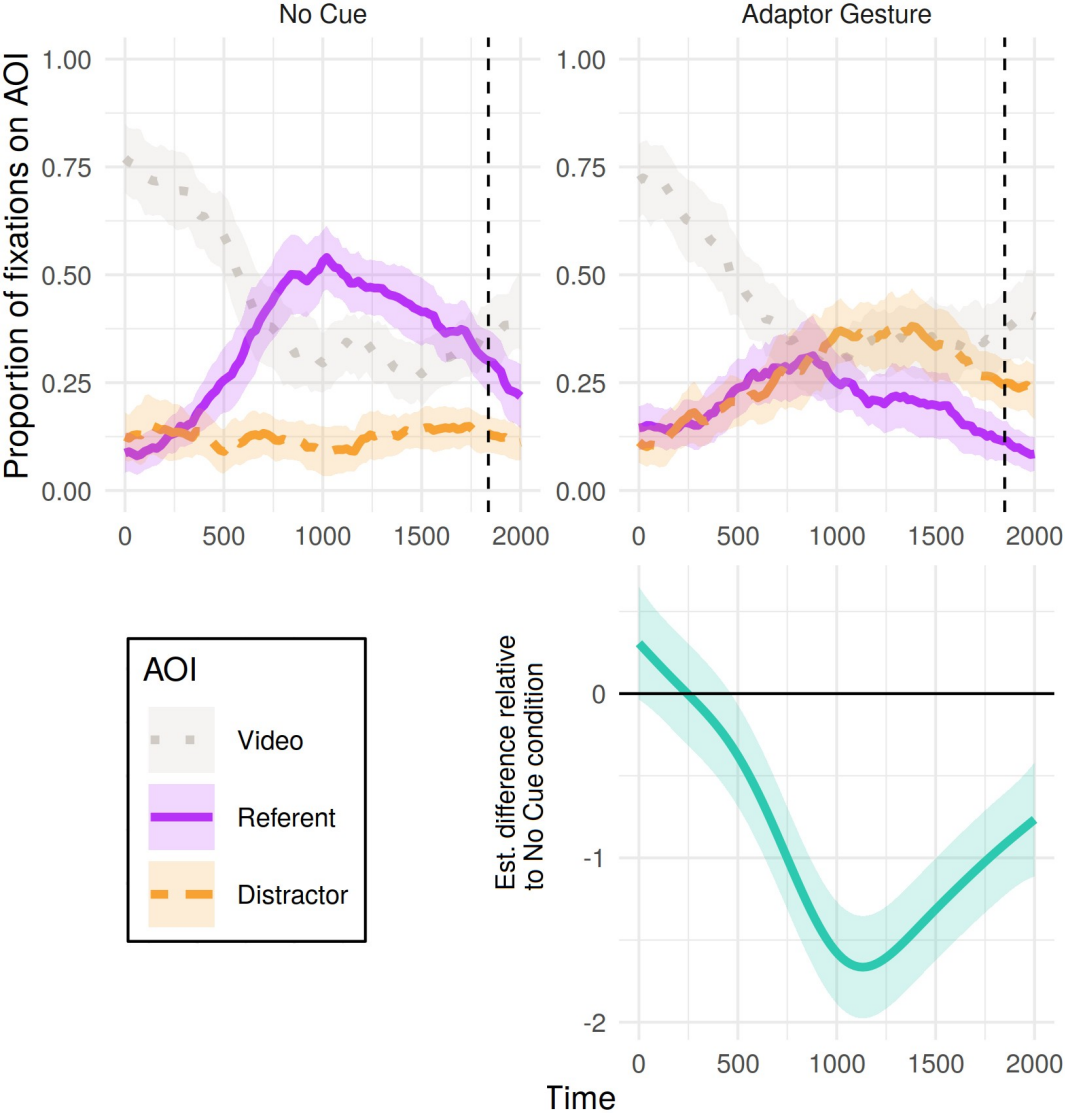
Eye tracking results for Experiment 2: Proportion of fixations to each object (referent or distractor) and the video, from 0 to 2000 ms post-referent onset, calculated out of the total sum of fixations for each 20 ms time bin. Shaded areas represent 95% confidence intervals derived via bootstrapping subject data (R = 1000). Bottom panel presents the estimated difference between non-linear smooths comparing each condition to the no-cue condition (negative value indicates fewer fixations to referent > neither > distractor in the cue condition). Pointwise 95%-confidence intervals are shown by the shaded bands—where these do not overlap with zero, the difference between conditions is significant.

Fig 7 shows the time course of fixations to referents, distractors and videos in critical trials for the 2000 ms from referent onset, split by presence of adaptor gesturing. Analyses conducted over this window of object (referent > neither > distractor) fixated in a given 20 ms revealed a reliable difference over time between conditions (see Table 11). The difference curve between conditions (Fig 7, bottom panel) indicates that from approximately 460 ms there was a greater probability of participants fixating away from the referent and towards the distractor in the adaptor gesture condition relative to the no cue condition.

**Table 11:** Eye-tracking model results, Experiment 2: Results from ordinal generalised additive mixed model of object fixated in a given bin (referent > neither > distractor).

*Parametric coefficients*	*Estimate*	*Std. Error*	*t-value*	*p-value*
(Intercept)	1.10	0.16	7.03	<.001
Adaptor Gesture	-0.89	0.15	-5.87	<.001
*Smooth terms*	*edf*	*Ref.df*	*F-value*	*p-value*
s(Time)	5.29	5.74	5.72	<.001
s(Time):Adaptor Gesture	6.48	7.56	124.72	<.001
s(Time, Participant)	128.55	179.00	92.31	<.001
s(Time, Item)	126.05	179.00	133.67	<.001
s(Participant, Condition)	33.43	38.00	23.56	<.001
s(Item, Condition)	31.76	38.00	12.65	<.001

### Mouse movements

**Fig 8:**
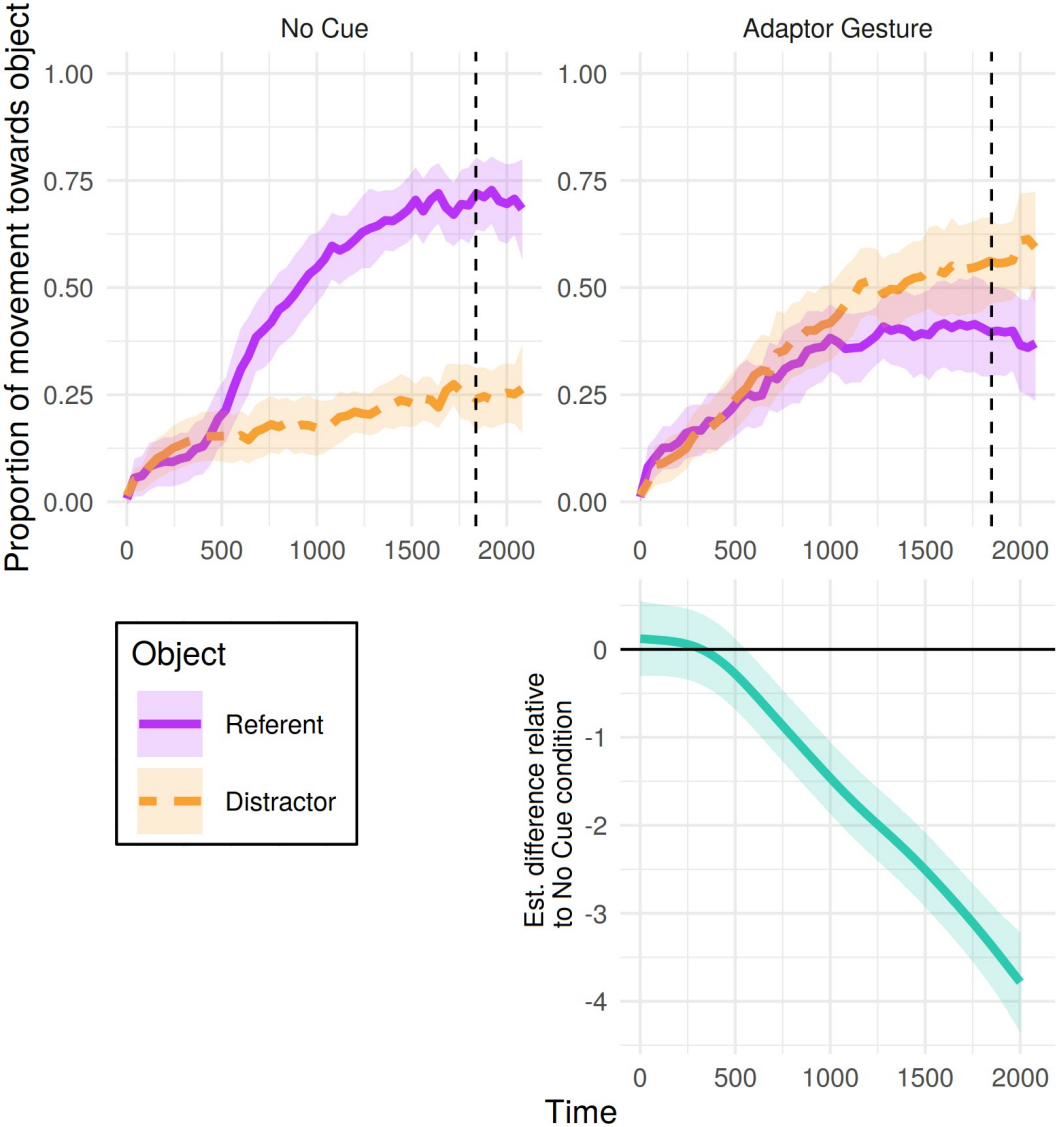
Mouse tracking results for Experiment 2: Proportion of cumulative distance travelled toward each object from 0 to 2000 ms post-referent onset. Proportions were calculated from the total cumulative distance participants moved the mouse until that time bin. Shaded areas represent 95% confidence intervals derived via bootstrapping subject data (R = 1000). Bottom panel presents the estimated difference between non-linear smooths comparing each condition to the no-cue condition (negative value indicates fewer mouse movements towards referent > neither > distractor in the cue condition). Pointwise 95%-confidence intervals are shown by the shaded bands—where these do not overlap with zero, the difference between conditions is significant.

Fig 8 shows the distance the mouse moved towards the referent and distractor over time, for 2000 ms from referent onset, split by condition. Patterning with participants’ fixations, analysis of the object (referent > neither > distractor) towards which the cursor travelled most cumulative distance over the course of this window revealed differences over time between conditions (see Table 12), emerging approximately 570 ms post referent-noun onset (Fig 8, bottom panel), and in the same direction (away from the referent and towards the distractor).

**Table 12:** Mouse-tracking model results, Experiment 2: Results from ordinal generalised additive mixed model of object towards which the cursor had moved most up to that time bin (referent > neither > distractor).

*Parametric coefficients*	*Estimate*	*Std. Error*	*t-value*	*p-value*
(Intercept)	1.14	0.28	4.05	<.001
Adaptor Gesture	-1.58	0.20	-7.85	<.001
*Smooth terms*	*edf*	*Ref.df*	*F-value*	*p-value*
s(Time)	3.02	3.32	5.46	<.001
s(Time):Adaptor Gesture	4.64	5.60	188.00	<.001
s(Time, Participant)	121.23	179.00	35.41	.160
s(Time, Item)	119.13	179.00	41.27	.578
s(Participant, Condition)	21.29	38.00	10.20	<.001
s(Item, Condition)	20.07	38.00	11.58	<.001

### Object clicks

Across the experiment, participants clicked on the referent in 53% of trials and the distractor in the remaining 47%. Table 13 shows the numbers of clicks to either object for each type of nonverbal behaviour (no cue vs. adaptor gesturing). As in Experiment 1, participants who viewed videos of a motionless speaker were more likely to click on the referent than the distractor (OR = 5.07, 95% CI = [2.92, 8.79], *p* < .001). The nonverbal behaviour shown in the video was found to influence participants’ judgements of deception: Relative to videos showing no cue to deception, those showing an adaptor gesture cue resulted in a reduced likelihood of clicking on the referent (OR = 0.06, 95% CI = [0.03, 0.12], *p* < .001, Table 14). There was no significant association between the presence of adaptor gesture and the times participants took to click the mouse (Table 15).

**Table 13:** Objects clicked, Experiment 2: Clicks recorded on each object (referent or distractor) split by condition (no cue vs. adaptor gesture).

	Clicks to Referent	Clicks to Distractor
**No Cue**	161 (80.9%)	38 (19.1%)
**Adaptor gesture**	48 (24.2%)	150 (75.8%)

**Table 14:** Objects clicked model results, Experiment 2: Mixed effects logistic regression model results of mouse clicks to referent over distractor.

*Predictor*	*Odds Ratio*	*95% CI*	*p-value*
(Intercept)	5.07	2.92–8.79	<.001
Adaptor Gesture	0.06	0.03–0.12	<.001
Var(1|Participant)	0.52		
Var(Adaptor Gesture|Participant)	1.44		
Var(1|Item)	0.15		

**Table 15:** Time-to-click model results, critical trials Experiment 2: Mixed effects linear regression model results of log transformed times taken to click the mouse.

*Predictor*	*Estimate*	*95% CI*	*t-value*
(Intercept)	7.47	7.38–7.57	155.23
Adaptor Gesture	-0.04	-0.12–0.04	-0.92
Clicked Distractor	0.09	0.01–0.16	2.17
Var(1|Participant)	0.03		
Var(Adaptor Gesture|Participant)	<0.01		
Var(1|Item)	<0.01		

## General discussion

In Experiments 1 and 2, we investigated the influence of a speaker’s nonverbal behaviour on judgements of deception, focusing respectively on trunk movements and on adaptor gestures. A recorded speaker referred to one of two objects as the location of some treasure. We manipulated the visual presentation of nonverbal cues while measuring listeners’ eye and mouse movements towards images of either the referent named by the speaker, or a distractor object. This allowed us to explore whether, and when, listeners began to associate nonverbal cues with deception.

Contrary to research on spoken hesitations [4], the results of Experiment 1 were not compatible with trunk movements eliciting effects similar to those of speech disfluency on listeners’ judgements of deception. The eventual object selected by listeners was only marginally affected by whether or not the video showed the speaker producing a trunk movement. The contrast of these findings with previous research [5] may reflect differences between beliefs about cues to deception (as indicated in questionnaires) and those cues which listeners associated with deception when presented with them. Alternatively, the inclusion of additional nonverbal behaviours in filler trials may have weakened the association between trunk movements and deception which has been found in previous research [3, 5]. This is partly supported by studies which found a facilitative effect of illustrative gesturing on listeners’ comprehension to be weakened for speakers who produce a lot of other, non-communicative movements [25]. Finally, evidence points to the importance of temporal synchrony in the integration of illustrative gesturing with speech [26]. In Experiment 1, trunk movements were presented before the onset of speech; this may have weakened any potential association between cue and interpretation.

Importantly, however, additional analyses of Experiment 1 suggested that other types of nonverbal behaviour used in filler trials (different static postures and adaptor gestures) were associated with judgements of dishonesty. The likelihood of participants clicking on the referent (an implicit judgement of truthfulness) was reduced following either of these cues; and eye tracking and mouse movement records suggest a stronger bias, especially following adaptor gestures, emerging early during the time-course of comprehension.

Experiment 2 was conducted to confirm the influence of adaptor gestures on judgements of deception in a study designed specifically to this end. Videos in Experiment 2 showed the speaker either producing a typically nervous adaptor gesture or sitting motionless. Results indicate a reliable association between adaptor gesturing and perceived dishonesty, as evidenced by the object selected. Furthermore, drawing parallels to research in speech disfluency [4], presence of adaptor gesturing was associated with changes in fixation and (mouse movement) preferences for objects in the display (with differences emerging after 460 ms and 570 ms in eye and mouse movements respectively).

It is important to note that our eye-tracking analyses do not allow us to determine whether fixation preferences were due to a bias toward the referent over the distractor, or one toward the referent over the video. In other words, our results could be attributed to differences in participants’ truth/lie interpretations between conditions, or differences in their visual attention (due to, for instance, attending to the video more in the cue compared to the no-cue conditions). However, visual inspection of the time-course of fixations suggests that differences in video fixations between conditions are small, hence it is unlikely that these were driving the difference in patterns of fixations we observed between conditions.

The studies presented here provide a visual-modality parallel with the findings from Loy et al. [4] which suggested that fluency of speech influences judgements of whether a speaker is lying. In keeping with Loy et al. [4], our results suggest that listeners may have an implicit bias to judge a speaker as honest in the absence of any obvious potential cue to deception—a trend which is present in other studies in deception detection [27, 28]. In both experiments, utterances presented with the speaker in a neutral posture and not gesturing biased listeners towards believing the speaker to be truthful, as shown by an increased tendency to fixate on, move the mouse towards, and eventually click on the object which was named by the speaker. Similarly to the effect of manner of spoken delivery on these judgements [4], the results here are compatible with the idea that manner of nonverbal delivery influences judgements of deception, in particular when the speaker is seen to produce typically anxious adaptor gestures alongside speech. Importantly, effects were detectable in the initial stages of linguistic processing, emerging in Experiment 2 during the same time window as that in which Loy et al. [4] found effects of speech disfluency, showing that the influence of visual cues on judgements of deception is not restricted to post-utterance reasoning.

Our findings are largely consistent with previous research on beliefs about, and judgements concerning, nonverbal cues to deception, suggesting that listeners perceive a range of nonverbal behaviours, both dynamic and static, as indicative of deceit [29, 30]. Additionally, the studies presented here indicate that the link between nonverbal behaviour and deception may be driven partly by those behaviours which the listener perceives as signalling anxiety in the speaker, although further research is needed to confirm whether this is the case. The lack of a reliable association between trunk movements and judgements of deception shows that care should be taken when generalising results on how people perceive deception across different nonverbal behaviours, as well as generalising from peoples’ beliefs about cues to deception [3, 5] to ‘live’ situations in which they are faced with a variety of possible cues. This is supported by the possible qualitative difference in looking behaviour between Experiments 1 and 2, with a flatter distractor curve in no-cue condition of Experiment 2 (Fig 7) compared to that of Experiment 1 (in which patterns are similar across conditions, Figs 3 and 5). This is perhaps a result of the larger variety of behavioural cues in Experiment 1 leading to ‘no-cue’ being less strongly associated with truthfulness (patterning with the mouse-clicks in no-cue conditions between experiments).

One important consideration of these experiments (as well as previous studies using this paradigm [4, 16]) is that participants are presented with a context in which speakers a) sometimes lie, and b) sometimes produce a behavioural cue. These cues are known to be believed to be indicative of lying (see [3]), but it is possible that some other cue may work just as well, or that the cues used here may in a different context be associated with something other than deception. In the context of the current task, participants reliably linked the presence rather than absence of adaptor gesturing to deception. It is questionable whether the same would be true if the only available cue was some random behaviour such as the speaker waving their hand above their head. The fact that participants did not so clearly associate other available cues with deception (e.g., trunk movements in Experiment 1), supports a view that with adaptor gestures specifically, listeners are detecting bodily cues for lying (rather than simply detecting any bodily cue). However, this remains an issue which could be clarified by future research.

The experiments presented here show that it is possible to extend the Visual World Paradigm to include visual information about the speaker, and not just the extensional world. By including a video recording of a speaker alongside recorded speech, it is possible to measure the influence of nonverbal behaviour on listeners’ online processing of the unfolding message, even when listeners eventually fixate other images in the display. This is perhaps because listeners are able to extract information about gestures through peripheral vision (see e.g. [31]). Overall, the studies here show that in utterance processing, the visual channel can have a rapid and direct effect on a listener’s pragmatic judgements, supporting the idea that communication is fundamentally multimodal: Speech and nonverbal behaviour interactively codetermine meaning.
